# Correlation analysis of aqueous humor metabolomics with myopic axial length and choroidal parameters

**DOI:** 10.1186/s12886-023-03101-1

**Published:** 2023-08-15

**Authors:** Jiechao Shao, Zongchan Zhang, Xuecheng Cai, Xinyu Wu, Baishuang Huang, Ye Shen, Jianping Tong

**Affiliations:** Department of Ophthalmology, First Affiliated Hospital, College of Medicine, Zhejiang University, Hangzhou, 310003 Zhejiang China

**Keywords:** Myopia, Aqueous humor, Metabolomics, Choroid

## Abstract

**Background:**

To explore differential metabolites in the aqueous humor of patients with different axial lengths and their correlations with axial length and choroidal parameters.

**Methods:**

In this study, we included 12 patients with axial lengths less than 24 mm, 11 patients with axial lengths between 24 and 26 mm, and 11 patients with axial lengths greater than 26 mm. We collected their aqueous humor samples during cataract surgery for liquid chromatography-mass spectrometry metabolomic analysis. Simultaneously, we collected relevant clinical parameters such as axial length, subfoveal choroidal thickness, and choroidal vascular index. Correlations between clinical data, differential metabolites, and clinical indicators were analyzed. In addition, we plotted receiver operating characteristic curves.

**Results:**

The results showed that axial length was significantly negatively correlated with choroidal thickness (r=-0.7446, P < 0.0001), and that several differential metabolites were significantly correlated with certain clinical parameters. After analyzing receiver operating characteristic curves, 5-methoxytryptophol and cerulenin were found to have excellent discriminative power, demonstrating their potential as biomarkers. In the enrichment analysis, we found that the differential metabolites among each group were involved in several special pathways (Taurine and Hypotaurine Metabolism, Vitamin B6 Metabolism, Pantothenate, and coenzyme A Biosynthesis), suggesting that abnormalities in these metabolic pathways may play a role in the process of axial myopia.

**Conclusions:**

Our study identified alterations in certain metabolic pathways in different axial lengths. At the same time, we found several metabolites with significant correlation with clinical indicators, among which 5-methoxytryptophol and cerulenin were associated with axial myopia.

**Clinical trial registration:**

Registration date:11/04/2022. Trial registration number: ChiCTR2200058575. Trial registry: The First Affiliated Hospital of the Zhejiang University School of Medicine.

## Background

Axial myopia is the most common type of myopia, and high myopia is defined as an axial length of > 26 mm. We focused our research on axial length, as it is one of the main causes of myopia. High myopia is not just a lengthening of the eye axis; it can even cause serious vision damage, such as cataracts, glaucoma, retinal detachment, macular fissure, and macular hemorrhage [1]. Methods to control myopia progression have shown varying results, including defocused spectacle lenses, orthokeratology, low-concentration atropine drops, and increased time spent outdoors [2–8]. It is expected that by 2050, nearly half of the world’s population will have myopia, and nearly one billion people will have high myopia [9]. From this perspective, the prevention and control of myopia has become a major issue in social and health care systems.

The main function of the aqueous humor (AH) is to maintain intraocular pressure; nourish the cornea, lens and vitreous humor; and carry away metabolic products from these tissues. Metabolites are located downstream of biological activity and have a strong correlation with phenotypes, such as myopia and axial length. Existing metabolomic studies on the aqueous humor of patients with myopia have advanced our understanding of the mechanisms underlying myopia. A project that used both capillary electrophoresis-mass spectrometry (CE-MS) and liquid chromatography-mass spectrometry (LC-MS) explored the aqueous humor collected from patients with high and low myopia and found more than 20 differential metabolites [10]. Another study used gas chromatography coupled with time-of-flight mass spectrometry (GC/TOF MS) to analyze 242 aqueous humor metabolites in patients with high myopia and controls, of which 29 metabolites were found to be significantly altered [11].

Cataract surgery is a highly mature surgical technique that can obtain both aqueous humor and preoperative eye-related data [12]. In this study, we collected data regarding the axial length (AL), anterior chamber depth (ACD), corneal endothelial cell count, subfoveal choroidal thickness (SFCT), and choroidal vascular index (CVI) of each patient. The aim of the present study was to analyze these clinical data in combination with metabolite results to expand the understanding of myopia and its prevention.

## Methods

### Subjects

The study enrolled 34 subjects and divided them into three groups: 12 in Group A, 11 in Group B, and 11 in Group C. Each group was distinguished by axial length, which was measured with an optical biometer. Group A had an axial length of less than 24 mm, group B had an axial length between 24 and 26 mm, and group C had an axial length greater than 26 mm. The inclusion criteria were patients who had cataracts that affected their normal life and work or required cataract surgery. The exclusion criteria were as follows: (1) History of ophthalmic surgery (including vitreous cavity injections). (2) Ocular diseases include glaucoma, uveitis, macular degeneration, retinal vascular diseases, diabetic retinopathy, etc. (3) Major systemic diseases such as tumors and hematological diseases, etc. (4) History of ocular trauma. The study protocol was reviewed and approved by the Clinical Research Ethics Committee of the First Affiliated Hospital, College of Medicine, Zhejiang University, China. All patients provided written informed consent.

### Clinical data measurement

All patients underwent a complete ophthalmological examination, including best-corrected visual acuity (BCVA), intraocular pressure (IOP), slit-lamp examination, fundus examination, optical biometer (OA2000, TOMEY CORPORATION), corneal endothelial examination (SPECULAR MICROSCOPE EM-4000, TOMEY CORPORATION), enhanced depth imaging OCT (EDI-OCT model, Spectralis OCT, Heidelberg), ophthalmic A-ultrasound, and B-ultrasound.

**Fig. 1:**
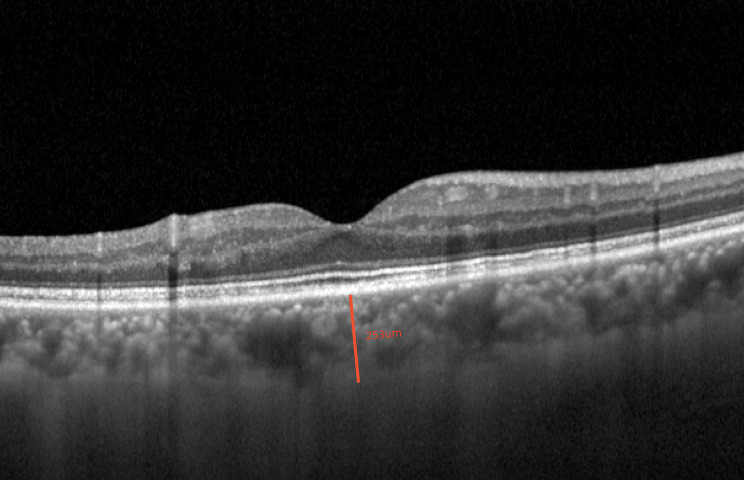
Measurement of subfoveal choroidal thickness

**Fig. 2:**
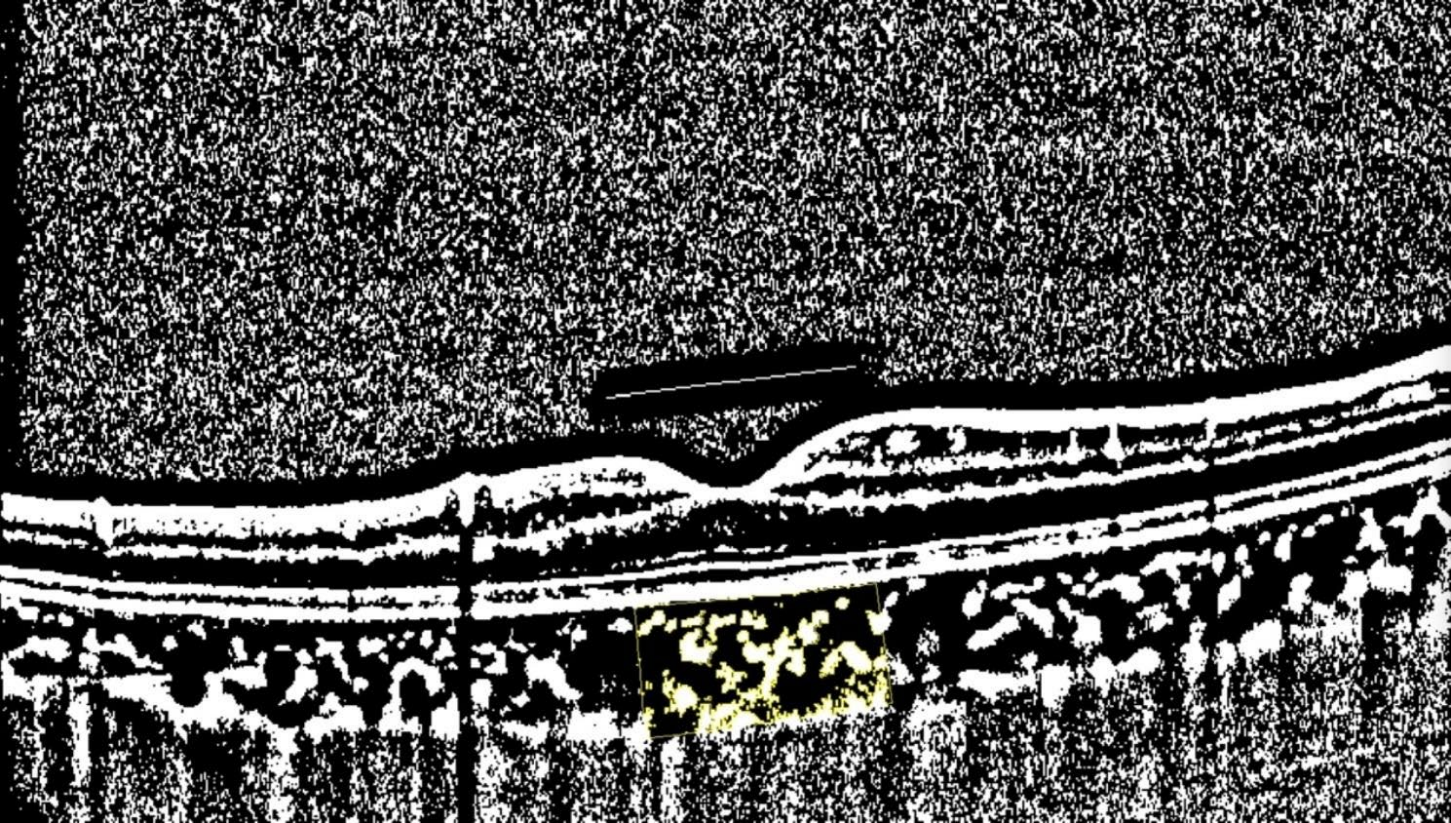
Binarization method with ImageJ software

AL and ACD were measured using an OA 2000. All OCT examinations were performed between 9 a.m. and 11 a.m. SFCT was defined as the distance between the subfoveal retinal pigment epithelium (RPE) outer edge and the choroid-scleral junction (Fig. 1). CVI was determined by binarizing and segmenting the OCT images according to the protocol described by Agrawal et al. [13]. The ImageJ software (http://imagej.nih.gov/ij, version 1.53 K) was used to binarize the OCT images. First, the image was converted into an 8-bit image and adjusted using the Niblack auto-local threshold method. The fovea was taken as the center, the range of 750 μm distance was determined on both sides, the polygon tool was used to select the total choroidal area (TCA) with a width of 1500 μm, which was then added to the ROI manager. The Color Threshold tool was used to select dark pixels as the luminal area (LA) to be added to the ROI manager (Fig. 2). The area of the white pixels is defined as the stromal area (SA). The CVI was calculated as the LA/TCA ratio. All measurements were repeated thrice by experienced ophthalmologists and were then averaged.

### Surgical sample collection

First, we confirmed the basic information of the patients. Then, the patients underwent a routine surgical procedure after receiving surface anesthesia. A cataract surgical incision was made and, approximately 100 µl of aqueous humor along the incision were collected as a surgical sample with a 1 ml sterile syringe. Finally, the sample was put into a sterile cryopreservation tube and numbered, before being quickly transferred and stored in a -80 °C refrigerator until use.

### Metabolites profiling and identification, metabolomic data analysis

The collected samples were thawed on ice. Metabolites were pretreated with 50% methanol buffer, and quality control (QC) samples were prepared. Samples were stored at -80 °C until formal analysis.

Metabolites were separated on a Thermo Scientific UltiMate 3000 HPLC. Compounds were separated using an ACQUITY UPLC BEH C18 column (100 mm*2.1 mm, 1.8 μm, Waters, UK) at 35 °C. The mobile phase comprised solvents A (0.1% formic acid in water) and B (0.1% formic acid in acetonitrile). The flow rate was 0.4 ml/min. The gradient conditions are as follows:0~0.5 min, 5% B; 0.5~7 min, 5–100% B; 7 ~ 8 min, 100% B; 8~8.1 min, 100–5% B; 8.1~10 min, 5%B.

A high-resolution tandem mass spectrometer Q-Exactive (Thermo Scientific) was used to detect metabolites. The instrument was operated in both positive and negative ion modes.

The acquired MS data was preprocessed using the XCMS software. Raw LC-MS data files were converted to mzXML format and then processed using the XCMS, CAMERA, and metaX toolboxes. Each ion was identified by combining retention time (RT) and m/z data. The metabolites were annotated by matching against the Kyoto Encyclopedia of Genes and Genomes (KEGG) and the Human Metabolome Database (HMDB). Simultaneously, we used an in-house fragment spectrum library of metabolites to validate metabolite identification.

The intensity of the peak data was further preprocessed using the metaX software. Features detected in less than 50% of the QC samples or 80% of the biological samples were removed, and the remaining peaks with missing values were imputed using the k-nearest neighbor algorithm. The data were normalized using Probabilistic Quotient Normalization (PQN). Quality control-based robust LOESS signal correction (QC-RLSC) was used to correct batch effects. Simultaneously, the Coefficient of Variation (CV) of all QC sample ions was calculated, and data with CV > 30% were excluded.

Differences in metabolites between the two groups were calculated using Student’s t-test and Fold Change (FC) values. We employed the supervised statistical method of Partial Least Squares Discrimination Analysis (PLS-DA) to assess metabolic differences between the groups. Volcano plots and enrichment analysis (based on the small-molecule pathway database SMPDB, https://smpdb.ca/) were performed for significantly different metabolites (while satisfying FC > 1.2 or FC < 0.833, P < 0.05) [14, 15].

### Clinical data analysis and correlation analysis with differential metabolites

Filtered by the following conditions: (1) trend calculation: we calculated the difference between the means of groups A and B. Then, we calculated the difference between the means of groups B and C. Next, we calculated the product of the two differences and took the part of the product > 0. (2) We calculated the FC value between each two groups. The FC that was > 1.2 or < 0.833 was selected. The differential metabolites screened according to the above steps were then subjected to Spearman correlation analysis with previously collected clinical indicators. Finally, a heat map was drawn. At the same time, Spearman correlations between several specific clinical parameters were also calculated. Several metabolites with significant correlations with clinical parameters were then selected and receiver operating characteristic (ROC) curves were drawn.

### Statistical analysis and graphing

Statistical data were analyzed using SPSS software (version 26). PLS-DA graph was drawn and differential metabolite enrichment analysis was performed using metaboanalyst 5.0 (https://www.metaboanalyst.ca/). Volcano plots and correlation graphs between clinical data were plotted using the GraphPad Prism software (version 9.40). The correlation heatmap of the clinical data and differential metabolites was drawn using Omicstudio’s cloud tool (https://www.omicstudio.cn/tool).

## Results

### AH sample results

In this study, we collected 34 AH samples with different axial lengths from patients undergoing cataract surgery. We collected 12 samples with AL < 24 mm (group A), 11 samples with AL between 24 and 26 mm (group B), and 11 samples with AL > 26 mm (group C), using 24 and 26 mm as the boundary. The basic information and clinical data for each group of patients are shown in the table below (Table 1).

**Table 1:** Basic and clinical data of the three groups of patients

characteristics	A	B	C	P value
number	12	11	11	
Eye (OD/OS)	8/4	7/4	6/5	0.826
age (mean ± SD)	63.667 ± 6.457	64.545 ± 9.234	59.091 ± 10.300	0.303
Gender (Male/Female)	5/7	4/7	3/8	0.768
AL (mean ± SD)	23.084 ± 0.565	24.639 ± 0.609	29.912 ± 2.268	<0.0001
SFCT (mean ± SD)	248.379 ± 71.639	201.520 ± 34.745	105.804 ± 66.474	<0.0001
CVI (mean ± SD)	65.259 ± 4.179	66.585 ± 2.970	67.311 ± 8.056	0.662
ACD (mean ± SD)	3.172 ± 0.328	3.199 ± 0.435	3.296 ± 0.229	0.662
Lens thickness (mean ± SD)	4.514 ± 0.401	4.515 ± 0.298	4.571 ± 0.318	0.905
Endothelial cells (mean ± SD)	2577.167 ± 219.337	2663.273 ± 143.632	2563.091 ± 207.433	0.430

### Clinical data results

**Fig. 3:**
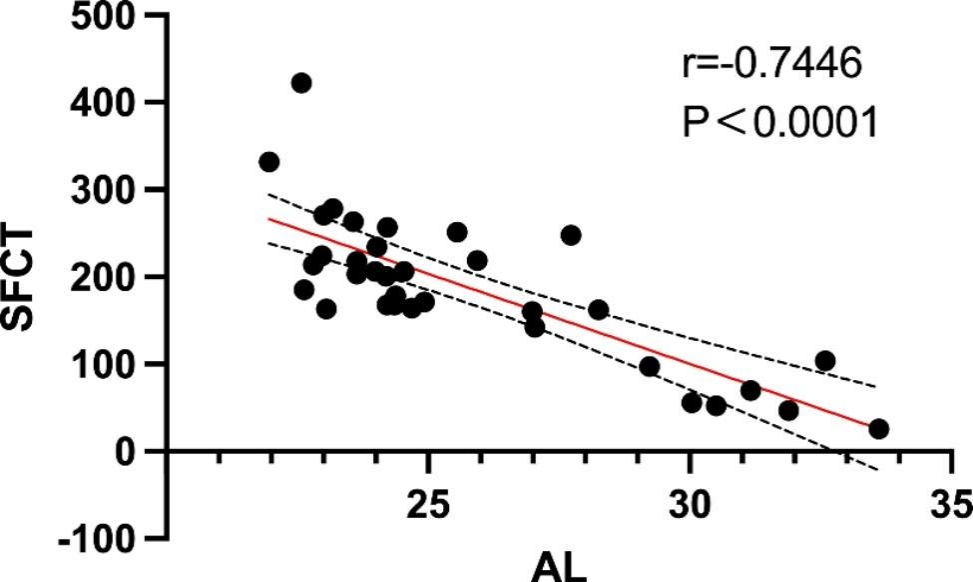
Correlation analysis between AL and SFCT

Correlation analyses of the AL, SFCT, and CVI were performed. Only AL was significantly associated with SFCT (r=-0.7446, P < 0.0001; Fig. 3). There was no significant correlation between AL and CVI or between SFCT and CVI.

### Metabolite results

A total of 1844 metabolites were identified in the positive ion mode, of which 620 were identified and named, 386 were annotated in the HMDB database, and 241 were annotated in the KEGG database. Meanwhile, a total of 2309 metabolites were identified in the negative ion mode, of which 357 were identified and named, 187 were annotated in the HMDB database, and 124 were annotated in the KEGG database.

**Fig. 4:**
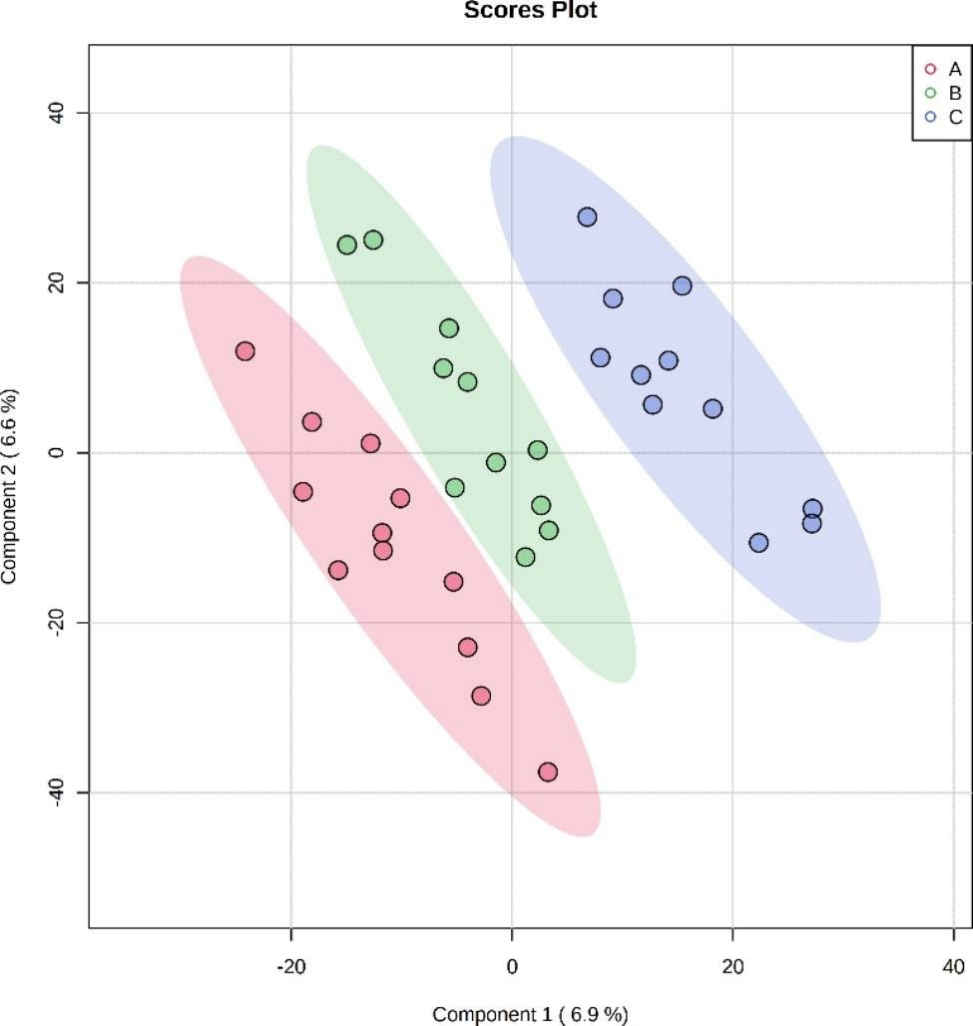
PLS-DA among the three groups (R2 = 0.99651, Q2 = 0.024885)

**Fig. 5:**
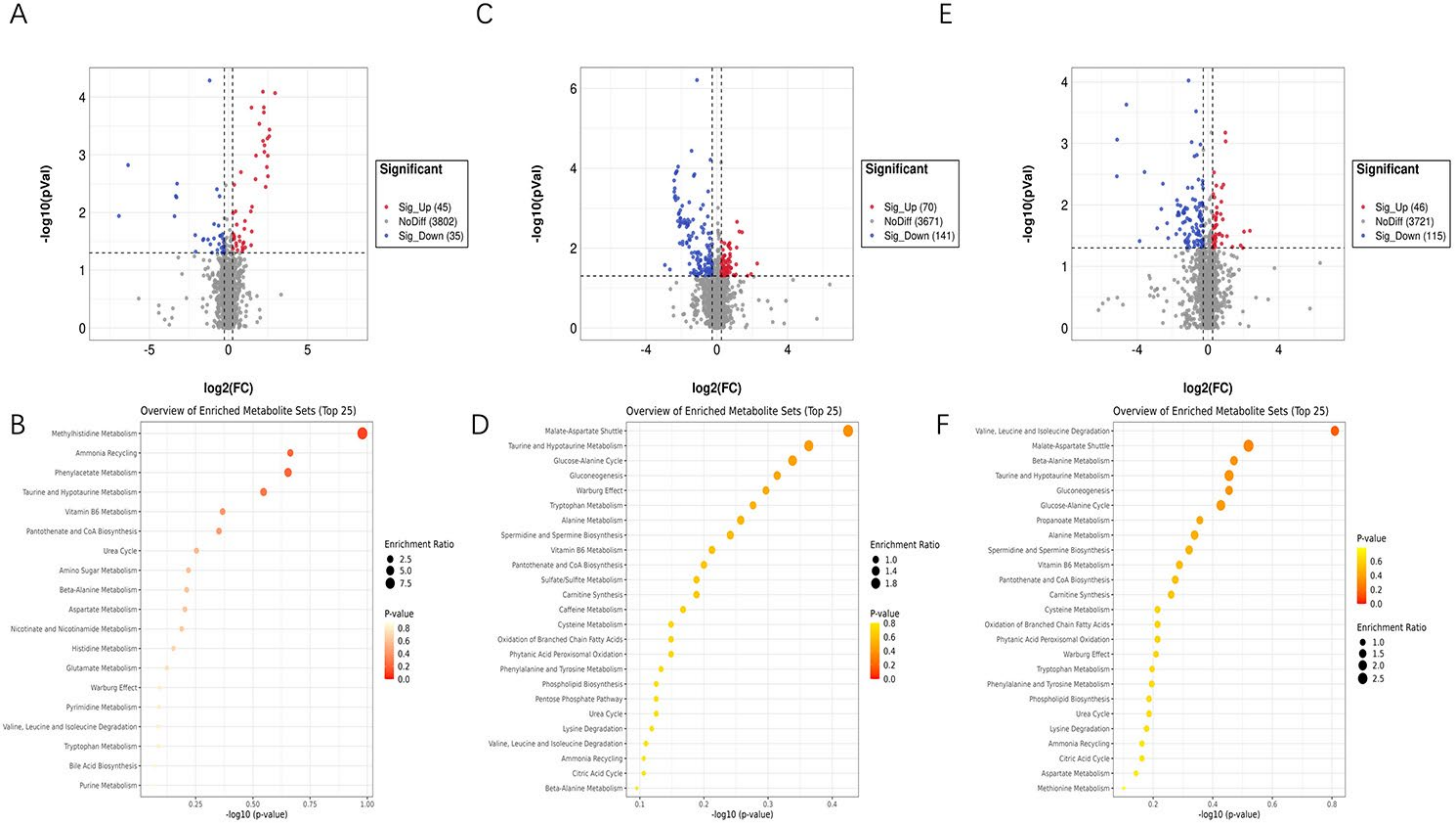
Volcano plot and enrichment analysis of differential metabolites Panels A and B represent the differential metabolite profiles of groups B and **A**. Panels C and D represent the differential metabolite profiles of groups C and **B**. Panels E and F represent the differential metabolite profiles of groups C and A

We employed the supervised statistical method PLS-DA to distinguish the groups so that metabolic differences could be identified assessed (Fig. 4). A specific threshold condition (P < 0.05, FC > 1.2 or < 0.833) was adopted and an enrichment analysis was performed for differential metabolites while plotting the volcano picture (Fig. 5). Between groups B and A, 80 differential metabolites were selected, of which 45 were upregulated and 35 were downregulated. These metabolites were mainly enriched in the following pathways: Methylhistidine Metabolism, Ammonia Recycling, Phenvlacetate Metabolism, Taurine and Hypotaurine Metabolism, Vitamin B6 Metabolism, Pantothenate and coenzyme A (CoA) Biosynthesis, urea cycle, amino sugar metabolism, beta-alanine metabolism and aspartate metabolism. Between groups C and B, 211 differential metabolites were selected, of which 70 were upregulated and 141 were downregulated. These metabolites were mainly enriched in the following pathways: malate-aspartate shuttle, Taurine and Hypotaurine Metabolism, glucose-alanine cycle, gluconeogenesis, Warburg effect, tryptophan metabolism, alanine metabolism, spermidine and spermine biosynthesis, Vitamin B6 Metabolism, Pantothenate and CoA Biosynthesis. Between groups C and A, 161 differential metabolites were selected, of which 46 were upregulated and 115 were downregulated. These were mainly enriched in the following pathways: valine, leucine and isoleucine degradation, malate-aspartate shuttle, beta-alanine metabolism, Taurine and Hypotaurine Metabolism, gluconeogenesis, glucose-alanine cycle, propanoate metabolism, alanine metabolism, spermidine and spermine biosynthesis, Vitamin B6 Metabolism, Pantothenate and CoA Biosynthesis. These three sets of contrasts jointly participate in the following pathways: Taurine and Hypotaurine Metabolism, Vitamin B6 Metabolism, Pantothenate and CoA Biosynthesis.

### Correlation analysis between clinical data and differential metabolites

**Fig. 6:**
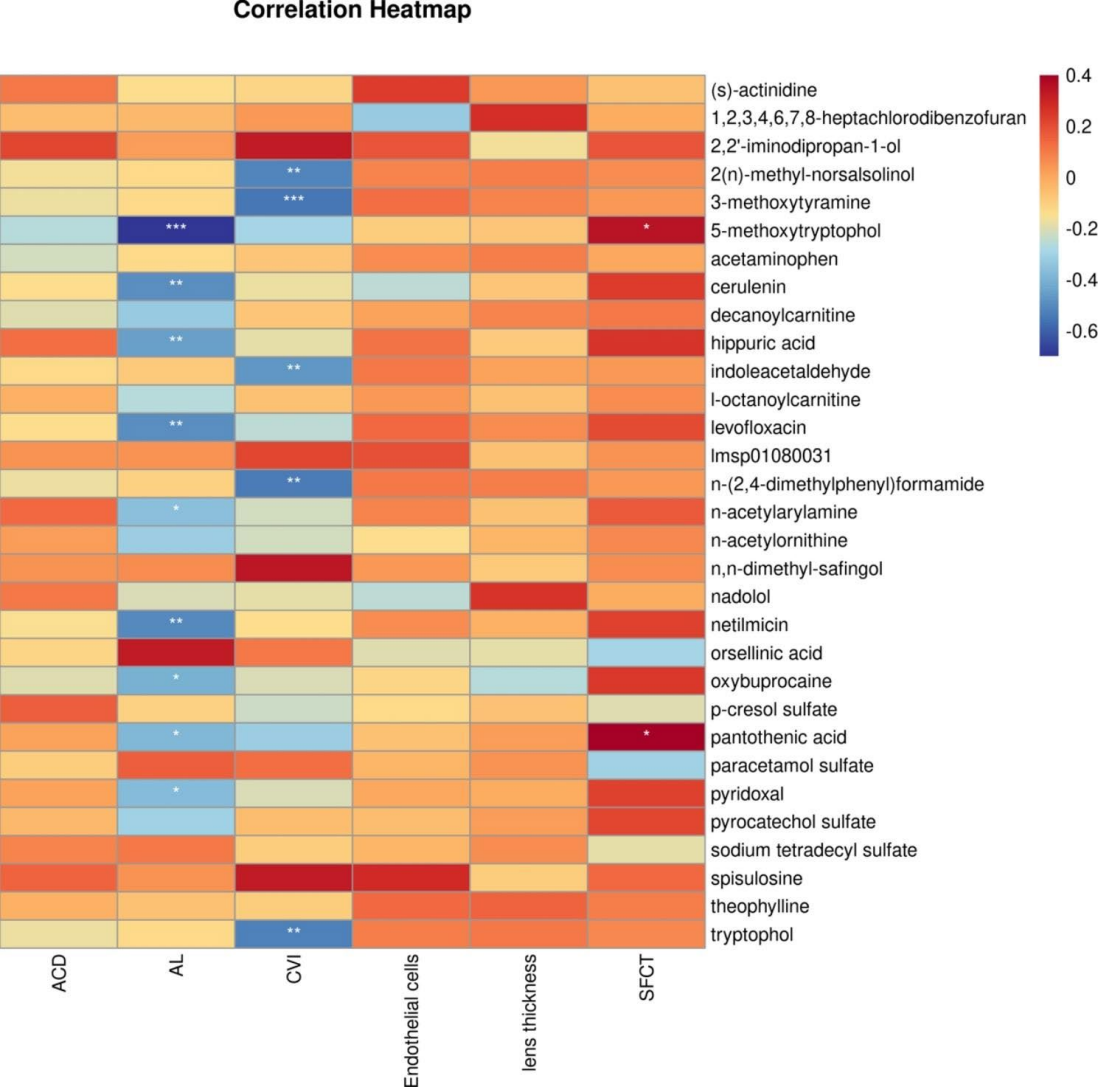
Heatmap for correlation analysis of clinical data with differential metabolites ***:P < 0.001, **:P < 0.01, *:P < 0.05, Red represents positive correlation, blue represents negative correlation. The darker the color, the stronger the correlation

Differential metabolites were determined based on trend analysis and FC values. Finally, 31 differential metabolites were screened and correlation analysis was performed with clinical data (Fig. 6). These metabolites did not correlate with ACD, endothelial cells, or lens thickness. AL was significantly and negatively correlated with the following metabolites: 5-methoxytryptophol, cerulenin, hippuric acid, levofloxacin, netilmicin, n-acetylarylamine, oxybuprocaine, pantothenic acid, and pyridoxal. The CVI was significantly and negatively correlated with the following metabolites: 2(n)-methyl-norsalsolinol, 3-methoxytyramine, indoleacetaldehyde, n-(2,4-dimethylphenyl)formamide, and tryptophol. Simultaneously, 5-methoxytryptophol and pantothenic acid were significantly positively correlated with the SFCT.

### ROC analysis

**Fig. 7:**
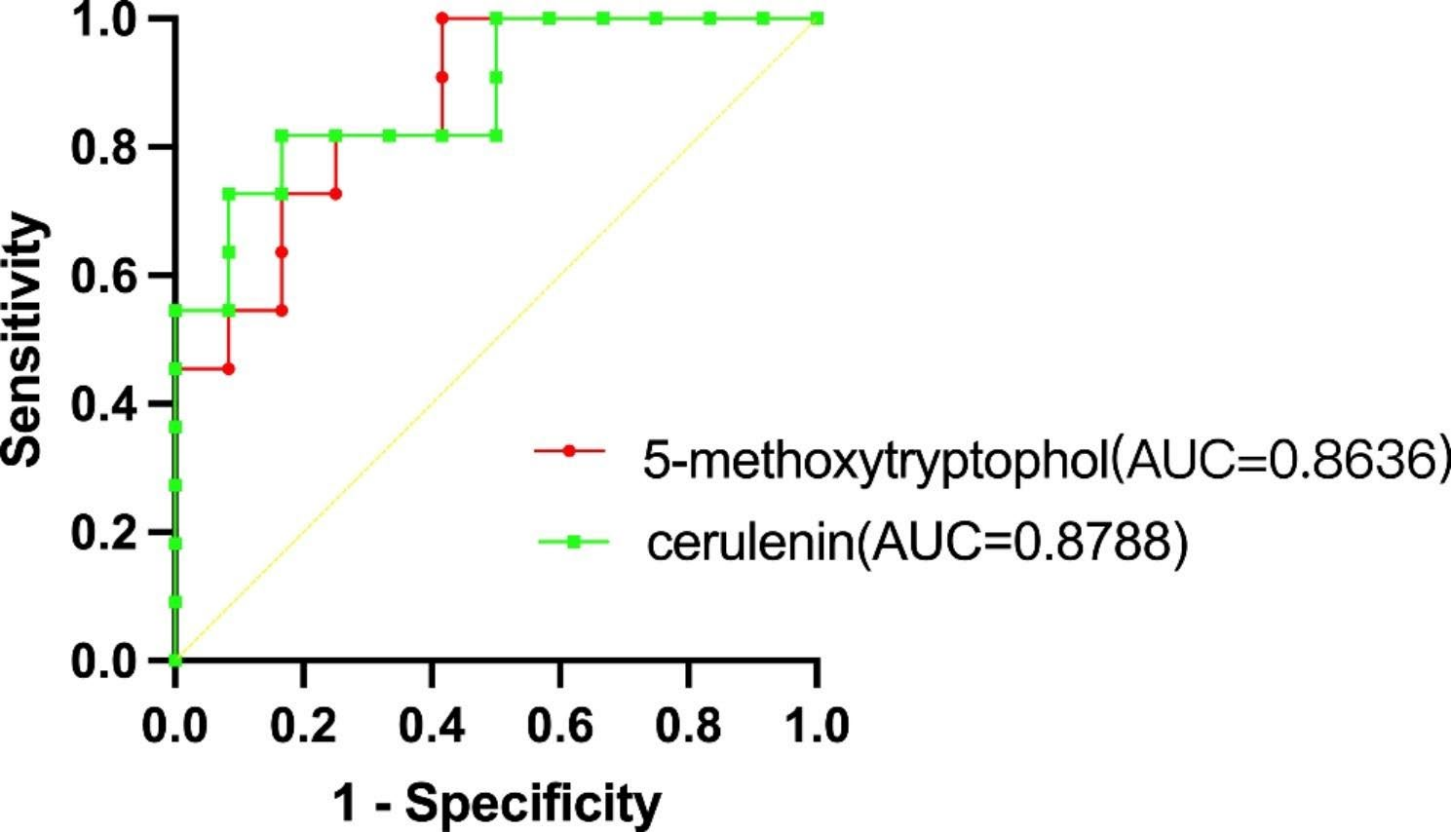
ROC curve used to distinguish group A from group B

**Fig. 8:**
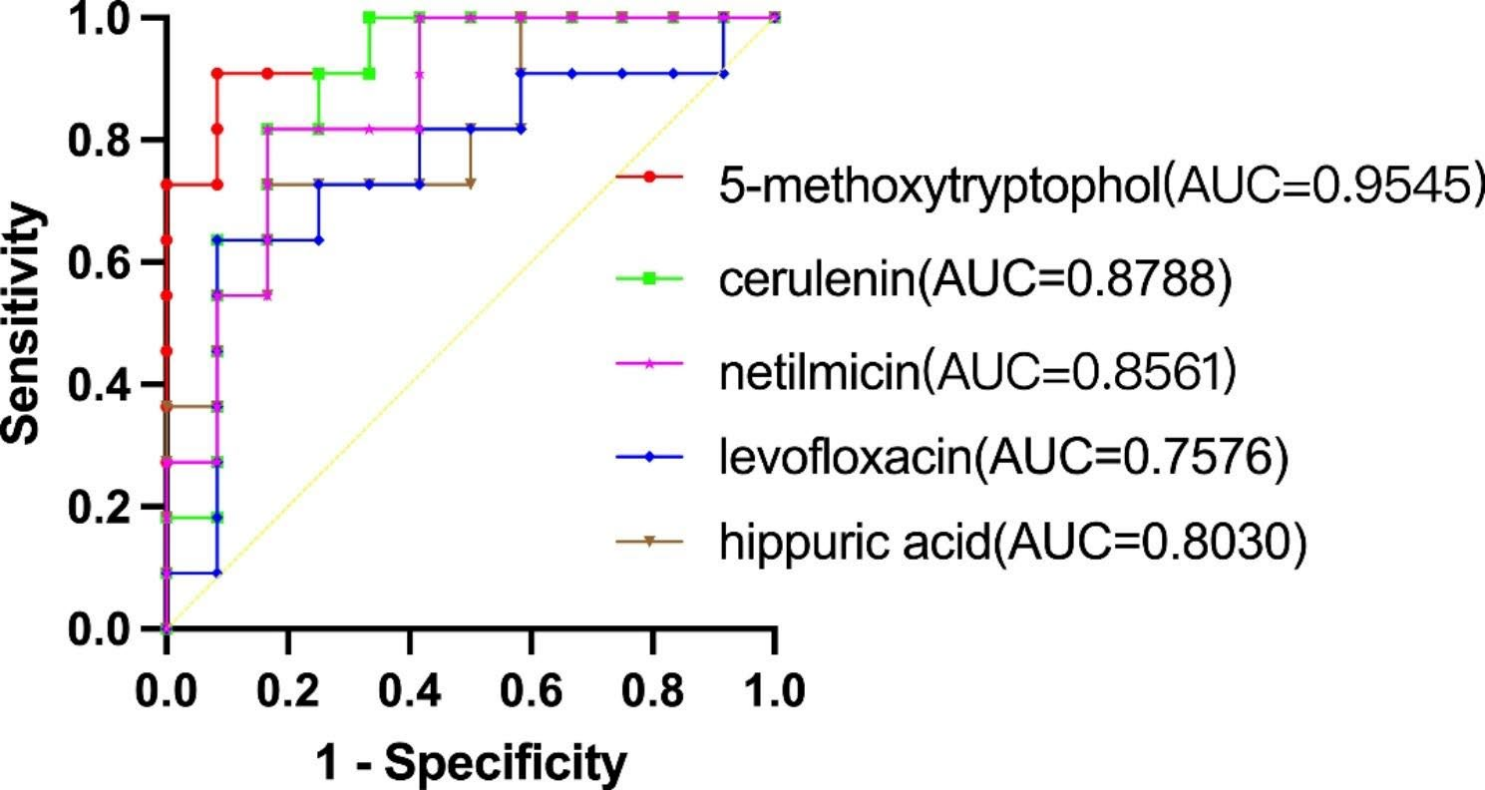
ROC curve used to distinguish group A from group C

We performed ROC analysis (correlation P < 0.01) and calculated the Area Under Curve (AUC) to further verify whether the differential metabolites that negatively correlated with AL could correctly distinguish group A from group B or group A from group C. Figure 7 shows that 5-methoxytryptophol (AUC = 0.8636, P = 0.0031) and cerulenin (AUC = 0.8788, P = 0.0021) have similar and good performance in distinguishing group A from group B. The following three metabolites have P-values greater than 0.05: netilmicin AUC = 0.6515, P = 0.2184; levofloxacin AUC = 0.5530, P = 0.6666; hippuric acid AUC = 0.5530, P = 0.6666. Figure 8 shows that the following five metabolites are feasible for distinguishing group A from group C: 5-methoxytryptophol AUC = 0.9545, P = 0.0002; cerulenin AUC = 0.8788, P = 0.0021; netilmicin AUC = 0.8561, P = 0.0038; levofloxacin AUC = 0.7576, P = 0.0364; hippuric acid AUC = 0.8030, P = 0.0138. Of these, 5-methoxytryptophol have the highest degree of discrimination, with the area under the curve reaching 0.9545.

## Discussion

In this study, we performed LC-MS metabolomic assays by grouping according to axial length, positive ion mode detected 1844 metabolites, of which 620 were named, and negative ion mode detected 2309 metabolites, of which 357 were named. In the comparison between groups B and A, 45 differential metabolites were upregulated and 35 were downregulated. In the comparison between groups C and B, 70 differential metabolites were upregulated and 141 were downregulated. In the comparison between groups C and A, 46 differential metabolites were upregulated and 115 were downregulated. In the mutual comparison of the three groups, it was found that the results of the enrichment analysis intersected several specific pathways (Taurine and Hypotaurine Metabolism, Vitamin B6 Metabolism, Pantothenate and CoA Biosynthesis), suggesting that some common metabolic pathways changed in different developmental stages of axial myopia.

Furthermore, we calculated the correlation among AL, SFCT, and CVI and found that only AL had a strong negative correlation with SFCT, which is consistent with previous reports [16, 17]. After trend analysis and limited FC values, we identified 31 differential metabolites and performed a correlation analysis with six clinical indicators. The results showed that only the AL, SFCT, and CVI were associated with these metabolites. The following metabolites were significantly and negatively correlated with AL: 5-methoxytryptophol, cerulenin, pantothenic acid, pyridoxal, levofloxacin, oxybuprocaine, hippuric acid, n-acetylarylamine, netilmicin. Among them, 5-methoxytryptophol and pantothenic acid were significantly positively correlated with SFCT.

5-methoxytryptophol is mainly synthesized by the pineal gland and produces certain fluctuations in diurnal changes in biological behavior. Previous studies have shown that 5-methoxytryptophol in the retina is mainly derived from melatonin conversion [18]. Meanwhile, 5-methoxytryptophol may be involved in controlling rhythmic photoreceptor metabolism [19]. In animal studies, researchers have found that 5-methoxytryptophol and melatonin are rhythmically synthesized in the pineal gland and retina, and that their synthetic trends in diurnal variation are opposite [20]. Studies have reported higher serum melatonin concentrations in myopic than in non-myopic patients, suggesting that light exposure and circadian rhythms play important roles in the development of myopia in humans [21]. The results of this study are consistent with our finding of a negative correlation between 5-methoxytryptophol and AL, indicating a non-negligible potential role of 5-methoxytryptophol in myopia. There is much evidence that circadian rhythm exists in the growth and development of the eyeball and the development of refractive error; therefore, whether the huge changes in the light environment of modern society and circadian rhythm disorders are related to changes in the levels of 5-methoxytryptophol and melatonin is worth exploring [22]. 5-methoxytryptophol had a significant negative correlation with AL and a significant positive correlation with SFCT, suggesting that 5-methoxytryptophol may be involved with factors such as choroidal blood supply in the development of myopia. This metabolite has the potential to be a powerful biomarker owing to its ability to discriminate group A from group B and group A from group C.

Cerulenin is a fatty acid synthase inhibitor that induces apoptosis in various tumor cells and is an antifungal antibiotic that inhibits fatty acid and steroid biosynthesis [23]. It does not appear that this metabolite is significantly associated with the development of myopia, but given its good differentiation between the group A, B and C, we believe that it has the potential to be a qualified biomarker. Further research into its role in the development of refractive errors is warranted.

Pantothenic acid, also known as vitamin B5, is required for CoA formation and fatty acid metabolism. CoA is a cofactor for a variety of enzymes in organisms and is involved in the metabolism of sugars, fats, proteins, and energy. Similar to pyridoxine, pyridoxal is a form of vitamin B6 that is involved in the metabolism of amino acids, such as glycine and serine, and acts as a cofactor. In an adult primate experiment, researchers found that pyridoxal protected retinal neurons from ischemic damage, suggesting that this metabolite plays a supporting role in retinal nutritional and metabolic processes [24]. In rats, diets lacking pantothenic acid and pyridoxine were found to cause changes similar to early vitamin A, isoleucine and valine deficiencies [25]. This result supports that with the growth of AL, choroidal thickness decreases, and the levels of pantothenic acid and pyridoxal also decrease, representing the continuous reduction in various nutritional metabolism levels during the process of myopia.

Levofloxacin belongs to a class of organic compounds known as quinoline carboxylic acids. Clinically, levofloxacin is mainly used for preoperative and postoperative anti-infection in ophthalmic surgery. Therefore, the detection of this product in the aqueous humor may be caused by local absorption. Oxybuprocaine is primarily used for surface anesthesia in ophthalmology; therefore, it can be detected within a reasonable range. However, their negative correlation with AL is puzzling. We believe that the increased eyeball volume in individuals with myopia may lead to a decrease in its relative concentration.

CVI is the ratio of the vascular components in the choroidal tissue. As AL increased, the SFCT gradually decreased. Although there was no statistically significant difference, the CVI showed an increasing trend. We believe that this is the result of the dynamic compensation of the eyeball for choroidal thinning during myopia. Our study found that 3-methoxytyramine, indoleacetaldehyde, tryptophol, 2(n)-methyl-norsalsolinol, n-(2,4-dimethylphenyl) formamide were significantly negatively correlated with CVI.

3-methoxytyramine is mainly found in the human brain, blood, and cerebrospinal fluid. A previous study found that the choroid thickness and level of 3-methoxytyramine in the vitreous increased in chicks under equiluminant artificial dynamic ON stimulation on a computer screen [26]. The result of the previous study is consistent with our results. As the CVI increases, it means a trend of decreasing SFCT, and the level of 3-methoxytyramine decreases. However, the causal relationship remains unclear and should be a target of future research.

In humans, indoleacetaldehyde is involved in many enzymatic reactions and tryptophan metabolism. Elevated levels of indoleacetaldehyde have been observed in Pseudoexfoliation syndrome (XFS), a disease associated with local stress or the development of inflammation that is usually associated with vasodilation and altered permeability [27]. Therefore, the level of indoleacetaldehyde also reflects the changes in the intraocular inflammatory microenvironment and vascular status. Tryptophol is derived from gut microbiota and has anti-inflammatory properties [28]. In this study, both indoleacetaldehyde and tryptophol were negatively correlated with CVI. High myopia is thought to be linked to the intraocular inflammatory microenvironment [29, 30]. Therefore, we believe that these two metabolites may be involved in changes in choroidal vascular composition through local inflammation, thus playing a role in the development of myopia.

Simultaneously, some differential metabolites were identified. Although they are significantly correlated with the above clinical indicators, owing to the current understanding of these substances is very little, their role in the development of myopia cannot be determined. Thus, future research may offer new perspectives. Hippuric acid, a combination of benzoic acid and glycine, is the main metabolite in the body and is a normal component of urine [31]. It is the most commonly used biomarker for biomonitoring occupational toluene exposure [32]. Netilmicin is an aminoglycoside antibiotic with an antibacterial effect similar to gentamicin. 2(n)-methyl-norsalsolinol has been identified as a putative endogenous neurotoxin in Parkinson’s disease patients [33]. The roles of n-acetylarylamine and n-(2,4-dimethylphenyl) formamide in aqueous humor require further exploration.

As each group of samples collected in this study was gathered during cataract surgery and the impact of cataract on the results was controlled, the influence of cataract on this study was relatively negligible. Furthermore, the samples for this study were obtained prior to the formal surgical procedure; therefore, the analysis of the samples and acquisition of clinical parameters were not affected by surgery.

We believe that the following shortcomings exist in this study: First, the number of samples in each group was not sufficient, which was due to our strict sample requirements, such as that patients cannot have hypertension or diabetes and that the samples must be obtained during surgery of the first eye. Second, if the metabolites in the vitreous humor can be simultaneously measured and combined with metabolite data in the aqueous humor and clinical data, there may be new insights that were not explored in the present study. Finally, multi-omic studies should be conducted as they may broaden our perspectives.

## Conclusions

In conclusion, our study identified alterations in certain metabolic pathways in different axial lengths. Through a joint analysis combining relevant clinical parameters, we found several metabolites with significant correlations, of which 5-methoxytryptophol and cerulenin had strong discrimination and were associated with axial myopia.

## Data Availability

The datasets used and/or analyzed in the current study are available from the corresponding author upon reasonable request.

## List of abbreviations

AH: Aqueous humor
CE-MS: Capillary electrophoresis-mass spectrometry
LC-MS: Liquid chromatography-mass spectrometry
GC/TOF MS: Gas chromatography coupled with time-of-flight mass spectrometry
AL: Axial length
ACD: Anterior chamber depth
SFCT: Subfoveal choroidal thickness
CVI: Choroidal vascular index
BCVA: Best-corrected visual acuity
IOP: Intraocular pressure
EDI-OCT: Enhanced depth imaging OCT
RPE: Retinal pigment epithelium
TCA: Total choroidal area
LA: Luminal area
SA: Stromal area
QC: Quality control
RT: Retention time
KEGG: Kyoto encyclopedia of genes and genomes
HMDB: Human Metabolome Database
PQN: Probabilistic Quotient Normalization
QC-RLSC: Quality control-based robust LOESS signal correction
CV: Coefficient of Variation
FC: Fold Change
PLS-DA: Partial Least Squares Discrimination Analysis
ROC: Receiver operating characteristic curves
CoA: Coenzyme A
AUC: Area Under Curve
XFS: Pseudoexfoliation syndrome
